# The PRO-GRESS model: A conceptual framework for gender-responsive reproductive health promotion among survivors of child marriage^☆^

**DOI:** 10.1016/j.dialog.2026.100297

**Published:** 2026-03-14

**Authors:** Erika Agung Mulyaningsih, Ismi Dwi Astuti Nurhaeni, Sapja Anantanyu, Anik Lestari, Haryani Saptaningtyas

**Affiliations:** Universitas Sebelas Maret, Surakarta, Indonesia

**Keywords:** Child marriage, Reproductive health, Gender, Violence, PRO-GRESS model

## Abstract

**Background/Purpose:**

Child marriage is a global problem affecting the health and well-being of women, especially in developing countries such as Indonesia. This study aims to develop a gender-responsive reproductive health promotion model to prevent violence against women survivors of child marriage, defined as girls who were married before age 18 and remain vulnerable to gender-based violence as a consequence of early marriage.

**Methods:**

Using an exploratory qualitative approach with a phenomenological design, the data were collected through in-depth interviews with seven female survivors of child marriage. Data were collected between late 2023 and mid-2024 who were married before age 18. Participants were selected using purposive sampling. The focus group discussion (FGD) was conducted with policymakers, and NGOs representatives in East Java, Indonesia, to capture policy and structural perspectives that support the development of the PRO-GRESS model.

**Results:**

Many women are trapped in a cycle of violence and reproductive health problems due to gender inequality and social pressures. The findings revealed key themes, including triggers for child marriage, the psychological experiences of victims, and social and structural barriers after marriage. The PRO-GRESS (Promotion of Gender Responsiveness for Reproductive Empowerment and Safe Sexual Health) model was developed as a framework for promoting gender-responsive reproductive health and preventing violence.

**Conclusion:**

This study suggests the need for cross-sector collaboration to address this issue comprehensively, involving policymakers, non-governmental organizations, and the wider community. These findings have important implications for the development of policies and programs that support women's empowerment and the protection of children's rights. Specifically, the PRO-GRESS Model offers a novel contribution by moving beyond the prevailing focus on the prevention of child marriage, and instead emphasizing the need to address survivors' post-child-marriage lives through empowerment, psychosocial recovery, and the provision of gender-responsive reproductive health services.

## 1 Introduction

Child marriage is a global problem affecting the health and well-being of women who marry before the age of 18. Every minute, 23 children are married before the age of 18 [1], [2]. This condition is a global problem that occurs in various countries, including Indonesia [3], [4]. In 2022, Indonesia received 52,396 marriage dispensation applications, with 50,748 decisions granted [5]. Girls are at seven times greater risk than boys [6]. Generally, they live in rural areas, with poor economic conditions, low education levels, gender inequality, and also because of unprotected sexual activity, which leads to unwanted pregnancies [4]. UNICEF data indicate that in 2018, the prevalence of child marriage was higher in rural areas (17.82%) than in urban areas (7.43%). The data further show that many girls who entered child marriage had limited educational attainment, with 33.94% having completed primary education and 44.86% having completed junior secondary education [6]. Child marriage is a violation of human rights and has various adverse effects, but this practice is still widespread. It is often considered a solution to economic problems, as well as unwanted pregnancies [7], [8].

Based on Law Number 16 of 2019 on the Age Limit for Marriage, both men and women are required to be at least 19 years old when registering their marriage. Data from the Ministry of Women's Empowerment and Child Protection of the Republic of Indonesia show that, from 2019 until the end of 2021, the number of early marriage cases in Indonesia increased by 30% annually. The Directorate General of the Religious Courts received approximately 34,000 applications for early marriage dispensation between January and June 2020. Of these applications, 97% were approved, with 60% involving marriages of girls under the age of 18 [5].

This condition shows that the existing regulations still have many loopholes for child marriage to occur. When they have obtained legal approval to carry out child marriage, the next problem is the impact that is already visible, but there has not been enough action to help those who have been trapped in child marriage. Therefore, what is needed is not just prevention but also to achieve significant change; solutions must be analyzed in a programmatic and layered manner [9].

In some areas, premarital pregnancies are difficult for women and their families, and they have to hide their pregnancies so that they do not receive adequate antenatal care, attempt illegal abortions, or go through pregnancies with poor health conditions and mental health problems [10]. The pregnancy caused them to receive severe social sanctions. Thus, marriage is considered a solution to the problem of social sanctions [11]. Their low education, knowledge, and skills mean that they miss out on many opportunities to work and find it difficult to break out of the cycle of poverty, as well as being vulnerable to violence and various reproductive health problems [7], [10]. The facts show that women who are survivors of child marriage are at increased risk of becoming victims of violence. The lower the age of the woman, the higher the risk of experiencing violence by her partner [12]. The data from WHO reveals that one out of three women has experienced violence in their lifetime [13]. The form of violence can be physical, sexual, or psychological. Violence that occurs against women due to child marriage is exacerbated by gender inequality and various societal norms prevailing in the community [14], [15]. WHO report indicates that social norms that accept violence perpetuate its cycle [16]. Thus, child marriage, which is also caused by gender inequality, increases a girl's risk of becoming a victim of violence [14], [17].

Violence against women often continues during pregnancy, and it can be perpetrated not only by intimate partners but also by their families. As many as 59% of women experience violence during pregnancy, a rate higher than that before pregnancy [18]. This violence includes physical, sexual, and psychological abuse and constitutes a serious public health problem due to its impact on reproductive health and the health of the newborn [14], [19], [20], [21], [22], [23], [24], [25], [26]. It has been reported that this violence increases the risk of maternal mortality, abortion, antenatal stress, and postpartum depression. It also poses a significant risk to the baby, increasing the incidence of prematurity, intrauterine fetal death (IUFD), low birth weight (LBW), and infant mortality [27].

Thus, it can be concluded that women who are survivors of child marriage are at risk of various impacts of gender inequality, which will have an impact on violence and lead to women's reproductive health problems [28]. Although numerous studies have examined prevention efforts, the fact is that the problem of child marriage is unresolved. Although it is known that they are a vulnerable group experiencing violence and various reproductive health problems, the fact is that there have been no preventive efforts so that women who have become survivors of child marriage do not fall further into a detrimental situation, because they will face the risk of violence and various reproductive health problems.

Therefore, a gender-responsive reproductive health promotion model is needed to prevent violence against women who are survivors of child marriage. In contrast to existing frameworks that predominantly emphasize the prevention of child marriage and its associated risk factors, this study introduces the PRO-GRESS Model, which centers on survivors' post-child-marriage experiences through empowerment, psychosocial recovery, and access to gender-responsive reproductive health services. This study aims to develop a gender-responsive reproductive health promotion model by exploring the perspectives of survivors, policymakers, and non-governmental organizations.

## 2 Methods

This study used an exploratory qualitative approach with a phenomenological design to understand the subjective experiences of survivors of child marriage deeply. This approach was chosen because it enables the exploration of the meaning that individuals attribute to a specific social event, including, the phenomenon of child marriage, which is often closely tied to cultural, social, and psychological dimensions.

This research was conducted in one of the districts in East Java. This district has been designated as a Child-Friendly District several times, but there are still problems, such as child marriage [29]. In addition, this region still faces a lower Gender Development Index (GDI) compared to the GDI of East Java Province and the National GDI.

Data collection was conducted through two main techniques: in-depth interviews and FGD. In-depth interviews were conducted with seven female survivors of child marriage. The interviews aimed to explore their personal narratives, perceptions, and life experiences in facing and undergoing child marriage. Informants were selected using purposive sampling and in strict accordance with ethical research principles. The inclusion criteria were female participants who had entered marriage before the age of 18, were survivors of violence related to child marriage, and had previously received case assistance from the Women's Crisis Centre (WCC) or the Office for Women's Empowerment and Child Protection.

The selection of participants was conducted with careful ethical consideration. First, potential participants were identified through the WCC and the Office for Women's Empowerment and Child Protection, as these institutions provide support to women who had formally reported their cases and received comprehensive assistance. All participants had completed the resolution of their cases, both in terms of legal processes and psychosocial recovery, and were therefore considered emotionally stable and willing to openly share their experiences for research purposes.

Second, initial willingness to participate was communicated by the survivors themselves through their case facilitators at the WCC or the Office for Women's Empowerment and Child Protection. This ensured that participation was entirely voluntary and not initiated directly by the researchers.

Third, in-depth interviews were conducted in the participants' homes. The researcher visited each participant together with a representative from the WCC or the Office for Women's Empowerment and Child Protection to ensure participants' comfort and sense of safety. Participants' prior relationships with these institutions functioned as an additional protective measure in case psychological distress emerged during the interview process. No participants experienced distress during or after the interviews.

Prior to each interview, the researcher explained the study objectives, procedures, principles of safety and fairness, confidentiality measures, and the voluntary nature of participation. Participants and their families were informed that they could withdraw from the study at any time if they felt uncomfortable. Modest inducements were provided as appreciation for participation. Each interview was conducted in the presence of a parent, and informed consent was obtained through signed consent forms by the participant, their parent, and an independent witness. Ethical approval for this study was obtained from the Health Research Ethics Committee of Poltekkes Kemenkes Semarang (Approval No. 1305/EA/KEPK/2023).

A total of seven participants were included in the final analysis. All participants were aged 15–17 years at the time of marriage and were aged between 17 and 18 years at the time of the interviews. All participants had completed junior secondary education, with two continuing their education at the time of data collection. Five participants resided in rural sub-districts, and two lived in semi-urban areas. All participants were divorced or separated at the time of the interviews.

Although nine potential participants were initially identified, data saturation was reached after seven interviews, as the eighth and ninth interviews did not yield substantively new themes. This aligns with previous methodological guidance indicating that thematic saturation in homogeneous samples is often achieved within six to twelve interviews.

Interviews were conducted between late 2023 until early 2024. The interview process was conducted in the local language (Javanese) and Indonesian, recorded, and then translated by a translator with a background as a lecturer in mental health nursing. Transcripts were translated from Javanese and Indonesian into English by bilingual researchers to ensure accuracy of meaning, especially for sensitive and psychologically related terms. Additionally, their background as mental health nurses provides the ability to better understand the informants.

Furthermore, data were collected through focus group discussion (FGD) attended by survivors, parents, academics, representatives from Religious Courts, Women's Empowerment and Child Protection Office, District Education and Culture Office, District Health Office, Ministry of Religion, and Academics, as well as NGOs from the WCC and Pesantren Srikandi Foundation. The FGD activity was held in mid-2024, at a higher education institution in East Java. The FGD aimed to obtain structural and policy perspectives on the phenomenon, as well as to confirm and enrich data from individual interviews. The process of selecting attendees is through an official letter addressed to the head of each institution, after which the head of the institution instructs the appropriate authorised parties to discuss the FGD topic.

Both in-depth interviews and the FGD were analyzed using the same thematic analytical approach developed by Braun and Clarke [30]. This approach was selected due to its relevance to the study's objectives, whereby in-depth interviews captured survivors' lived experiences, while the FGD was interpreted to elucidate structural dynamics and cross-sectoral policy perspectives.

The analysis process was conducted through six stages: (1) *familiarization with the data* through re-reading interview and FGD transcripts; (2) *initial coding* by identifying units of meaning from participant quotes; (3) *grouping of codes into initial themes*; (4) *reviewing and refining themes*; (5) *naming and defining themes*; and (6) *writing thematic narratives* containing the researcher's interpretation and supporting quotes. By identifying the main themes that emerged from the interview transcripts and discussions and developing patterns of relationships between themes, a conceptual model was formulated that represents the social and structural dynamics of the child marriage phenomenon.

To ensure the validity of the data, this study employed the triangulation of sources and verification of findings, adhering to the principle of *trustworthiness* in qualitative research [31]. Triangulation was conducted by comparing the results of in-depth interviews with seven informants of child marriage victims and the results of FGD involving cross-sector policymakers (health, education, legal, and social). This aims to enrich perspectives and confirm emerging thematic patterns.

Additionally, an ongoing process of researcher reflexivity was employed, including the recording of memos during the coding process to minimize interpretive bias. Findings were also reconfirmed through a limited *member-checking* process, where some quotes or interpretations of themes were returned to informants for clarification and agreement. The analysis documentation, in the form of code tables, theme maps, and selected quotes, was systematically collated as part of the *audit trail* to ensure transparency of the analysis process and traceability of the relationship between raw data and thematic interpretations.

## 3 Result

Through thematic analysis, five major themes were identified that illustrate the continuum from the determinants of child marriage to women's empowerment and resilience. This research yielded interconnected themes, describing the lived experiences of female survivors of child marriage, their obstacles, environmental pressures, and hopes. These themes emerged from participant narratives and were enriched by FGD with policymakers and representatives of non-governmental organizations. These findings highlight how early marriage affects psychological well-being, reproductive independence, and access to social protection and support systems.

### 3.1 The following is the model generated based on the analysis:

#### 3.1.1 Theme 1: context and triggers of child marriage

One of the primary triggers for child marriage in this study was unwanted pregnancy in adolescence. These pregnancies often occur outside of marriage and prompt families to quickly marry off girls to avoid social disgrace or community pressure. In most cases, the decision to marry is made not based on the child's awareness or readiness but rather in response to family pressure and social norms.

“I wasn't ready to get married, but when I was three months pregnant, my parents insisted I must. They said it was better to marry than to bring shame to the family in front of our neighbours.” *(Informant 1, married at 16 after experiencing an unwanted pregnancy).*

The decision to marry as a result of an unwanted pregnancy puts girls in a very vulnerable situation. Not only do they have to face a change in identity as wives and mothers, but they are also mentally and physically unprepared for these roles.

Additionally, all informants reported that their partners were significantly older, with a difference of more than 5 years. This age gap creates unequal power dynamics, where the male party often holds complete control in a dating relationship.

He was 23 when we got married, I was 16. He said he wanted to be responsible, but he neglected me, even when I was pregnant (*Informant 3, married age 16, husband age 23).*

This unequal relationship often results in a lack of equal communication, dominance in decision-making, and, in some cases, violence. Unwanted pregnancies and age disparities are a dangerous combination in creating unhealthy and unfair marital situations.

#### 3.1.2 Theme 2:physical and psychological experiences during early marriage

Child marriage often leaves a profound psychological impact on informants. Most stated that they were not ready to accept the reality of pregnancy and the role of motherhood. The pregnancy phase is often accompanied by emotional rejection of the condition of the body and the future baby due to coercion in the relationship and a lack of psychological readiness.

“When I found out I was pregnant, I cried every night. I could not accept it. It felt like all the dreams were destroyed.” *(Informant 4, married age 15 years).*

However, this psychological dynamic is complex. After the baby was born, some informants began to accept the presence of the child and tried to carry out the role of mother more stably, although still accompanied by feelings of anxiety and fatigue.

“When the baby was born, I was confused and afraid. However, over time, I truly came to love my child. He was the only encouragement, I was even willing to busk on the side of the road to get money when my baby ran out of formula milk, I was not ashamed, as long as to could get money, I would do anything for him (baby), and I also did not care anymore even though my husband never provided a living.” (*Informant 2, married at the age of 16).*

In addition to ambivalent feelings towards pregnancy, most informants experienced psychological and physical violence from their partners. This form of violence includes insults, intimidation, and physical violence.

“I was almost killed once, he pointed a knife at my neck, but my father found out, so I was saved.” (*Informant 6, married at the age of 15).*

This experience indicates deep psychological trauma and highlights the vulnerability of girls in early marriage, which is shaped by gender-based social norms and unequal power relations that continue to limit survivors' autonomy and social standing after child marriage.

#### 3.1.3 Theme 3: social and structural barriers in living life after child marriage

The impact of child marriage is not only felt personally but also in the form of social and structural barriers. Economic hardship is a common experience experienced by informants. Without adequate work skills and education, they are financially dependent on their partners or parents.

“I want to work, but I am confused about what job to do because I have not graduated from high school.” (*Informant 5, married at the age of 16).*

This economic condition is exacerbated by limited access to education. One informant was even forced to drop out of school after her pregnancy was discovered, while others tried to survive through the catch-up package program.

“The school contacted me and offered me the choice of dropping out or being expelled from school. In the end, I chose to drop out, even though I still wanted to go to school.” *(Informant 1, married at the age of 15).*

“I joined the catch-up package, even though sometimes I could not attend because I had to take care of my child. However, I still wanted to continue.” (*Informant 3, married age 17).*

In addition, almost all informants experienced reproductive health problems. They did not receive adequate ANC (Antenatal Care) services, had difficulty breastfeeding, and admitted that they did not understand baby care well.

“At that time, I was sick and had diarrhoea for several days, but I did not want to be checked by a midwife because I was afraid that I would be found out to be pregnant because my period was 3 months late.” (*Informant 7, married at the age of 15).*

#### 3.1.4 Theme 4: protective factors and social support

Despite facing various pressures and limitations, some informants noted that the presence of social support was a crucial factor in maintaining self-resilience. Parents who continued to accept them, as well as companions from NGOs or local communities, provided a sense of security and encouragement to help them survive.

“If it was not for my mother, I might have given up. My mother helped take care of my baby, while my father left the house for several months because he was embarrassed by the neighbours” (*Informant 2 married at the age of 16*).

“There was a lady from the foundation who often came, she helped convey it to my parents and helped take me to the midwife.” (*Informant 6 married at the age of 16*).

This support shows that an empathetic and supportive social environment acts as a protector from the more severe negative impacts of child marriage.

#### 3.1.5 Theme 5: hope, agency, and pathways to independence

Although many informants were trapped under challenging conditions, some demonstrated hope and agency in their efforts to escape the situation. The hope for a better future motivated them to work, study, and fight for an independent life.

“Now I work in a restaurant. I do not busk anymore, so I have a salary and money for my children and mother.” (*Informant 3, married age 16).*

“Now I have taken care of my passport, and the plan is to invite my work friends to go abroad because it is difficult to find work here.” (*Informant 7, married at the age of 15).*

This narrative shows that not all survivors of child marriage remain in a passive position. Agency and resilience emerge from personal motivation, supported by access to information, training, and a passion to improve the fate of children.

### 3.2 FGD results: cross-sectoral perspectives on CHILD Marriage

Table 1 summarizes the key perspectives expressed by participants during the focus group discussion.

**Table 1:** FGD report.

No	Participants	Number	Information
1	Religious Courts	1	1. Understanding regulations and laws is crucial for citizens to understand their rights as both human beings and citizens. 2. Prevention of child marriage is important. When families plan to apply for a marriage dispensation, they prepare documents to enable the Religious Court to grant a marriage dispensation permit. This condition presents a dilemma for the judge because the answers provided by the applicant and the documents are complete, so the existence of evidence is the basis for the judge to issue a marriage dispensation decision.
2	NGOs	2	1. Women who are victims of violence have the right to report and receive assistance provided by the Population Control and Family Planning, Women's Empowerment and Child Protection Service, or NGOs^a^ such as WCC.^b^ Assistance is invaluable for victims because psychological issues and children's helplessness, which women and their families frequently encounter, prevent them from solving problems appropriately. The existence of help provides knowledge and reinforcement, enabling victims to make informed decisions based on complete information. 2. Involve the village government in preventive efforts. 3. No child should be forced to quit or be expelled from school, as everyone has the right to an education. 4. Adolescents should be involved in a discussion about contraception as knowledge, and the discussion should be conducted thoroughly to provide them with an in-depth understanding.
3	Women's Empowerment and Child Protection Office	2	1. It is agreed that assistance has a positive impact on victims. 2. FGD participants expressed differing views regarding the practice of marriage dispensation. Those who supported marriage dispensation argued that it serves to protect the fetus and to ensure that the child attains a legitimate and socially recognized civil status.
4	District Education and Culture Office	2	1. The education department needs to make efforts to convey reproductive tools and sex education to school children through formal channels, beginning at the lowest level. 2. Sexual education is needed through peer counsellors and school health efforts because there has been no evaluation of peer counsellors to date.
5	District Health Office	2	1. Access to mentoring and strengthening during teenage pregnancy must be opened, and it is necessary to see them as a vulnerable group that not only has a high risk of pregnancy but must be seen as a whole in terms of bio-psycho-social-spiritual aspects. 2. Mentoring can be conducted through existing devices, such as the Family Support Team, which comprises cadres, village midwives, and other community members.
6	Ministry of Religion	1	Educating parents is crucial for strengthening the bond between children and their parents, especially for expectant mothers. 1. The media should promote an understanding of the rights and obligations of husbands and wives. 2. It is essential to understand religion as a foundation for behaviour. 3. The importance of building gender equality at the family level.
7	Academics	2	Academics play an important role in conducting research and community service in the field of health promotion and gender equality, especially in terms of preventing child marriage and violence against women. The involvement of academics can influence policy in a region.

**Source**: Primary Data FGD, 2024.

a NGO = Non-Governmental Organizations.

b WCC = Women Crisis Centre.

The results of the FGD presented in Table 1 highlight the significant roles of various stakeholders in addressing child marriage and its consequences. The Religious Courts emphasized the legal aspects and the challenges faced in granting marriage dispensations, while NGOs such as the WCC and Pesantren Srikandi Foundation stressed the importance of psychological and social support for victims. Furthermore, the District Health Office and the District Education and Culture Office reinforced the necessity of reproductive health education and peer counselling initiatives to prevent early pregnancies and their subsequent complications. These discussions indicate a collective acknowledgement of the issue and a commitment to implementing targeted interventions to protect young girls from the adverse effects of child marriage.

The conceptual model in this study was developed through the integration of empirical findings from the field and theoretical interpretation. Primary data were obtained through in-depth interviews with seven female informants who were survivors of child marriage and violence, as well as FGD involving policymakers from various agencies, such as the Health Office, District Education and Culture Office, Religious Court, Ministry of Religion, NGOs, and the Women's Empowerment and Child Protection Office.

Thematic analysis was conducted manually, following the six phases by Braun and Clarke (2006): familiarization, initial coding, theme searching, reviewing, defining, and reporting. Two researchers independently coded and discussed emerging patterns to enhance inter-coder reliability.Thematic analysis of the interviews produced consistent patterns of experience, such as social pressure due to unwanted pregnancy, age inequality between couples, gender-based violence, economic difficulties, and limited access to education and reproductive health services. On the other hand, several informants showed dynamics of resistance and recovery through social support, alternative education, and economic empowerment efforts. These findings were confirmed and further enriched through FGD, during which stakeholders highlighted the weak protection system, low access to reproductive services, and the absence of a comprehensive prevention approach.

Based on the integrated analysis of in-depth interviews and FGD, the researchers developed a thematic map to illustrate how the key themes and sub-themes are interconnected.

**Fig. 1:**
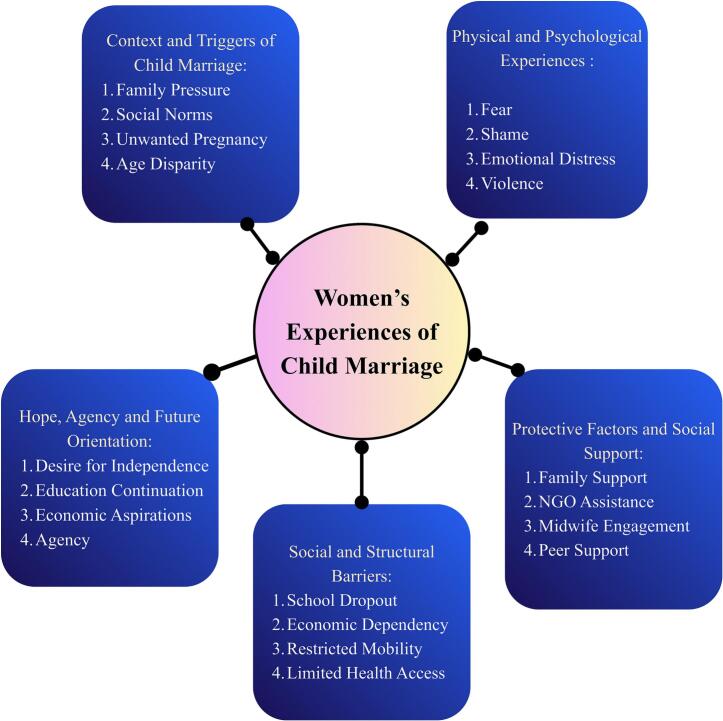
Thematic map of the experiences of women survivors of child marriage.

**Fig. 2:**
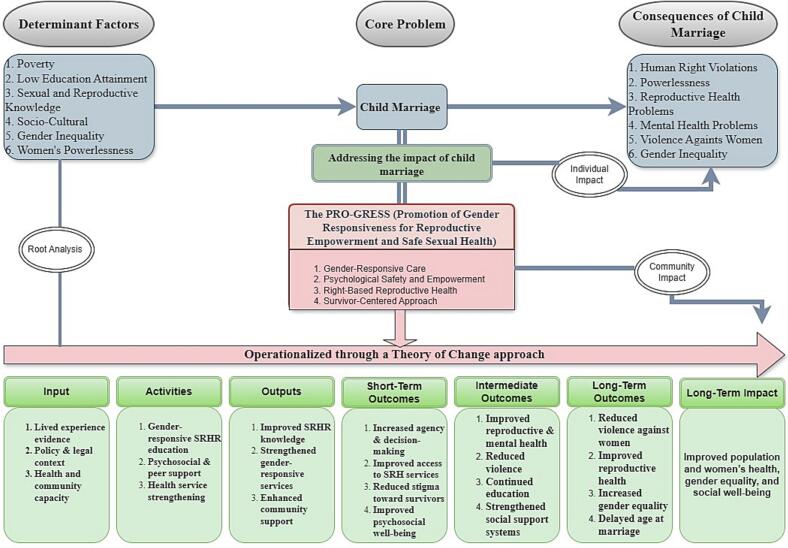
PRO-GRESS model: gender-responsive reproductive health promotion framework for survivors of child marriage. SRHR refers to sexual and reproductive health and rights.

The thematic map (Fig. 1) interrelated themes, including triggers of child marriage, psychological experiences, social-structural barriers, protective factors, and resilience pathways. Arrows represent causal and reinforcing relationships among themes. A cross-theme analytical interpretation shows that psychological trauma expressed through fear, shame, and emotional suppression is not merely an individual reaction but is reinforced by structural gender norms that prioritize obedience, silence, and endurance. This pattern aligns with socio-ecological evidence showing that gendered social norms operate at multiple levels, including familial and community contexts [32]. In particular, family pressure, consistently identified in both interviews and FGD in this study, functions as a mechanism of social control that restricts girls' agency, normalizing early marriage as a “solution” to economic hardship and concerns about premarital relationships [33]. These interlinked dynamics reflect a pathway in which structural constraints generate emotional consequences, which in turn limit decision-making power, forming the basis for systematic inequality [34]. This analytical pathway provides the conceptual foundation for synthesizing the PRO-GRESS Model as an emergent, data-driven, and theory-informed framework, developed inductively from the interviews and FGD. (See Fig. 2.)

Based on these findings, the researcher developed a different approach and described the causal flow and impact of child marriage, as well as opportunities for intervention. This model is built on a theoretical approach that combines the Theory of Change (ToC) as the basis for social change strategies and Gender Theory as a lens for understanding unequal power relations in victims' experiences.

The PRO-GRESS (Promotion of Gender Responsiveness for Reproductive Empowerment and Safe Sexual Health) model was developed to explain the complex dynamics between the causes, impacts, and strategies for addressing child marriage at the individual and community levels. This model integrates empirical research findings with a Theory of Change approach, which maps the causal relationships between social and structural conditions, survivors' experiences, and gender-based empowerment efforts [11], [32]. The model's primary objective is to strengthen gender-responsive reproductive health promotion through a survivor-centered approach.

Root cause analysis shows that interrelated factors are the result of interactions, including poverty, low education, limited reproductive health knowledge, socio-cultural norms that normalize early marriage, gender inequality, and women's empowerment [35], [36]. The interaction of these factors creates conditions of vulnerability that trigger child marriage as a core problem.

Child marriage has various serious consequences, both at the individual and community levels. Impacts on individuals include human rights violations, loss of independence, reproductive and mental health problems, and the risk of domestic violence [37], [38]. At the community level, this practice reinforces gender inequality and structural poverty, ultimately hindering the achievement of social welfare. These multiple impacts emphasize the need for a comprehensive strategy that focuses not only on prevention but also on the care and empowerment of survivors.

The Theory of Change approach is used to operationalize the PRO-GRESS model through a chain of change consisting of inputs, activities, outputs, and short-, medium-, and long-term outcomes. Initial resources (inputs) include survivors' lived experiences, the policy context, and community capacity [39]. Activities include gender-sensitive reproductive health education, psychosocial support, and strengthening health services. These activities produce outputs in the form of increased reproductive health knowledge, more gender-responsive services, and strengthened community support.

In the short term, this model is expected to increase women's agency and decision-making capacity, expand access to reproductive health services, reduce stigma against survivor, and improve psychosocial well-being. Intermediate outcomes include improved reproductive and mental health, reduced violence, and continued education and social support. In the long term, the implementation of the PRO-GRESS model is expected to reduce violence against women, increase gender equality, delay the age of marriage, and improve the health and social well-being of women and the population as a whole.

This model is used to solve the problem of child marriage; one factor, such as poverty, has many negative consequences for the reproductive and sexual health of girls. These consequences threaten the health of mothers and their babies, including problems such as death during childbirth, physical and sexual violence, isolation, depression, cervical cancer, and the risk of sexually transmitted diseases [11]. Pregnant girls are at high risk of premature birth and higher neonatal mortality than other women. Early marriage of girls can increase the risk of sexual and reproductive complications [40]. As stated in the background, the incidence of child marriage is still very high, and marriage is often considered a solution to problems such as poverty or unwanted pregnancies. However, children who have experienced marriage are faced with various problems that so far have not been specifically attempted to prevent.

This model comes with a ToC approach and involves multiple sectors, including policymakers and NGOs [41], [42], [43]. The need for cooperation is unavoidable because the impacts include various aspects that touch on human rights issues, powerlessness as women, reproductive health issues, mental health issues, violence perpetrated by partners, and also gender inequality [8], [38]. Key interventions include strengthening the legal framework, increasing access to education for girls, including those concerning human rights and reproductive rights, involving religious leaders and other sectors such as the media, and utilizing technology for awareness campaigns [44], [45].

Interventions with the ToC approach need to be planned strictly by involving various influential parties, adjusting to local conditions, involving copyists, and mobilizing support from the community under the health promoter's supervision to increase success in the problem of violence against women and child marriage [46]. The ToC concept helps health promoters in communities prioritize what needs to be done, as well as the time and parties involved [43], [47]. This concept helps map out so that cross-sector collaboration has a role and supports each other for the same goal.

## 4 Discussion

Based on the research results, a health promotion model was developed that addresses the problems of vulnerable groups that have not received attention so far. Many studies and policies are focused on preventing child marriage. At the same time, the facts on the ground show that marriage proposals before the age of 19 are still approved due to various underlying factors, especially unwanted pregnancies. The promotion of reproductive health through a gender-responsive approach is carried out by community health workers. Health workers (for example, midwives) have a significant role as promoters of reproductive health. [22], [48], [49], [50] The presence of midwives as workers in the community allows victims to be known, found, and given health services, especially related to reproductive health [51], [52] Health service providers must understand that they tend to hide or even end their pregnancies in dangerous ways, so cooperation is needed to be able to know their whereabouts and provide the best services as part of the right to health services [53], [54].

Health workers, such as midwives, need to look at the problem of child marriage more comprehensively, not just about medical diagnoses such as girls who are pregnant with anemia, Chronic Energy Deficiency, or Cephalopelvic Disproportion [55]. Also, to see the individual girl who is pregnant in its entirety, facing social, economic, and environmental pressures and a lack of knowledge. Based on the informants' experiences, although some of them refused to go to the midwife because they were worried that their pregnancy would be discovered, they believed that the midwife was the most appropriate person to help them deal with difficulties during pregnancy, inability to breastfeed, and care for the baby. The midwife was contacted at important moments in the victim's life and was considered a health worker who understood what was happening to help women regain their abilities [56], [57].

Thus, it is hoped that this model will serve as a campaign to prevent various problems associated with child marriage. Health workers in the community are not able to do it alone, so a multi-sector approach is essential [58], [59]. Through the Department of Education, efforts can be made to campaign on reproductive health, the Department of Religion can socialise the dangerous impacts of child marriage, and the Department of Health, through its policies, can strive to ensure that children who are already pregnant still get optimal health services. Health promotion must be carried out to increase awareness among the community, parents, and religious leaders, empowering girls in this case.

This framework is used to understand the dynamics of powerlessness and resistance experienced by women survivors of child marriage. Resources, agency, and achievements can only be realised through cross-sector cooperation. Child marriage is driven by multiple and complex factors and should not be understood merely as a cultural practice or an individual decision, but rather as a structural phenomenon rooted in unequal power relations, patriarchal gender norms, and broader social inequalities. The experiences of survivors illustrate that their post-child-marriage vulnerabilities are shaped by social and institutional structures that constrain autonomy, limit access to reproductive health services, and restrict opportunities for psychosocial recovery. To broaden the understanding of the structural and institutional context, this study also draws on the Social Relations Approach, which examines gender relations within five central institutions: the household, community, market, state, and civil society [60]. This approach helps to interpret the systemic inequalities revealed through cross-sectoral FGD. The framework is based on several theoretical ideas [61].

The ToC approach through multisector collaboration will complement each other to achieve the prevention of violence against women who have become survivors of child marriage, as they are a vulnerable population. Midwives as health workers in the community have the opportunity to be health promoters in the community [57]. Almost all pregnant women need midwife services to carry out Ante Natal Care (ANC), and this provides an opportunity for midwives to provide reproductive health education, gender equality, and prevention of violence [19], [62], [63]. Through programs in the community that involve policy makers, synergy is possible by paying attention to the needs of the local community directly [64].

Thus, this model not only represents the micro and macro situation of victims but also provides conceptual direction in efforts to promote reproductive health based on rights and gender equality. This model is compiled based on thematic results from in-depth interviews with seven informants who are survivors of child marriage and one FGD session with cross-sector stakeholders. Themes such as gender inequality, domestic violence, and reproductive health vulnerability appear repeatedly in the victim narratives. Meanwhile, the FGD reveals how the public service, legal, and education systems have not been able to respond effectively to this condition.

The findings of this study indicated that survivors of child marriage experienced physical and psychological violence, as well as reproductive health problems. At the policy and service level, survivors should be treated in accordance with the Istanbul Protocol (UN), an international guideline for documenting, investigating, and reporting cases of violence to support accountability and access to justice [65]. In the Indonesian context, legal and service pathways are available through Law No. 23/2004 on the Elimination of Domestic Violence and Law No. 12/2022 on Sexual Violence Crimes, as well as through Integrated Service Centres and medical–forensic verification procedures such as visum et repertum. Although forensic psychiatric evaluation was not conducted in this study, it is recommended as part of comprehensive survivor-centered services in the future, as it can objectively assess the psychological impact of violence, strengthen legal documentation, and inform the design of appropriate recovery interventions [66].

## 5 Limitation

The limitations of this study stem from the fact that the informants went into child marriages due to premarital pregnancy, which subjected them to social sanctions from the community. The findings are based on participants from a specific geographical area, which may limit their applicability to other settings. However, the causes of child marriage can vary by regions, such as poverty or cultural norms that condone the practice of child marriage.

Additional limitations include the small sample size and reliance on a single focus group, which may limit the generalizability of findings. Moreover, the translation process from Javanese and Indonesian into English may have introduced subtle interpretation bias despite rigorous review. Recruitment was also restricted to survivors already assisted by protection services, possibly excluding more marginalized cases.

## 6 Conclusion

The survivors of child marriage in this study face interconnected challenges, including unwanted pregnancies, gender-based violence, economic dependence, and limited access to education and reproductive health services. The PRO-GRESS model offers a gender-responsive, cross-sectoral framework to address these issues through empowerment, legal protection, and community-based health promotion. Implementation of this model can guide policymakers, health workers, and civil society organizations in developing integrated interventions that uphold women's rights and improve reproductive health outcomes. Although survivors of child marriage often face negative perceptions regarding out-of-wedlock pregnancies, every individual deserves opportunities for empowerment, protection, and a better future.

The PRO-GRESS model offers a novel conceptual contribution by integrating gender responsiveness, reproductive rights, and psychological resilience into a unified framework for post–child-marriage health promotion. Unlike previous models that primarily focus on prevention or service provision, PRO-GRESS highlights the empowerment continuum among women survivors, from vulnerability to agency, within social and structural contexts. Future research could further test and adapt this model in other cultural or regional settings with larger and more diverse samples to explore its applicability and scalability.

### 6.1 Research contribution

This research has provided significant theoretical and practical contributions in the field of reproductive health promotion and protection of female survivors of child marriage. From a theoretical perspective, the development of the PRO-GRESS Model enriches the treasury of qualitative-based conceptual frameworks that integrate victim perspectives with the Theory of Change and gender theory approaches. This model can be used as a reference for cross-sector interventions that are responsive to reproductive health needs and protection from gender-based violence.

Practically, this research offers concrete policy recommendations for the government, educators, health workers, and civil society organizations. The results of the FGD showed that cross-sector collaboration was still partial and reactive. Therefore, this model also functions as a strategic guide in the development of promotive-preventive programs that are transformative, based on children's rights and gender justice.

The PRO-GRESS model can be integrated into national and regional reproductive health programs, serving as a supporting framework for women who have survived child marriage. This model is also relevant for cross-sectoral gender mainstreaming strategies and community-based protection systems, as it promotes psychosocial recovery, empowerment, and gender-responsive services. Therefore, PRO-GRESS can serve as an implementation guide for policymakers and health service providers.

## Statement on the use of generative AI and AI-assisted technologies in the manuscript preparation process

During the preparation of this manuscript, the authors used ChatGPT (OpenAI) and Grammarly to assist with translation and grammar checks. After the manuscript was drafted, the authors used the assistance of a certified proofreader to double-check the English language. The authors reviewed and edited the content as needed and take full responsibility for the content of the published article.

## CRediT authorship contribution statement

**Erika Agung Mulyaningsih:** Writing – original draft, Investigation, Conceptualization. **Ismi Dwi Astuti Nurhaeni:** Methodology, Conceptualization. **Sapja Anantanyu:** Validation, Data curation. **Anik Lestari:** Project administration, Formal analysis. **Haryani Saptaningtyas:** Writing – review & editing, Resources.

## Ethical approval statement

This study has been ethically cleared and issued an ethical certificate from Poltekkes Kemenkes Semarang, No 1305/EA/KEPK/2023. All participants and their guardians signed written informed consent forms. Confidentiality and anonymity were ensured through pseudonyms, and participation was voluntary. A distress protocol was established, and psychological support was available through the WCC if needed.

## Declaration of competing interest

The authors declare that they have no known competing financial interests or personal relationships that could have appeared to influence the work reported in this paper. This publication did not receive any specific grant from funding agencies in the public, commercial, or not-for-profit sectors.
